# Integrated Strategy for Low-FODMAP Bread Formulation: Selection of Wheat Flours and Microbial Starter

**DOI:** 10.3390/foods15162792

**Published:** 2026-08-09

**Authors:** Fernanda Galgano, Antonella Tramutola, Nicola Condelli, Angela Lomonaco, Teresa Scarpa, Rocco Casale, Tiziana Di Renzo, Stefania Nazzaro, Marco Cintoni, Maria Cristina Mele, Isabella D’Antuono, Anna Reale

**Affiliations:** 1 Department of Agricultural, Forest, Food and Environmental Sciences, University of Basilicata, Via dell’Ateneo Lucano 10, 85100 Potenza, Italy; antonella.tramutola@unibas.it (A.T.); nicola.condelli@unibas.it (N.C.); angela.lomonaco003@unibas.it (A.L.); teresa.scarpa@unibas.it (T.S.); rocco.casale@unito.it (R.C.); 2 Department of Agricultural, Forest and Food Sciences, University of Turin, Via Verdi 8, 10124 Turin, Italy; 3 National Research Council, Institute of Food Sciences (CNR-ISA), Via Roma 64, 83100 Avellino, Italy; tiziana.direnzo@isa.cnr.it (T.D.R.); stefania.nazzaro@isa.cnr.it (S.N.); 4 Clinical Nutrition Unit, Department of Medical and Surgical Sciences, A. Gemelli University Polyclinic Foundation IRCCS, Largo A. Gemelli 8, 00168 Rome, Italy; marco.cintoni@unicatt.it (M.C.); mariacristina.mele@unicatt.it (M.C.M.); 5 Centre for Research and Training in Human Nutrition, Catholic University of the Sacred Heart, 00168 Rome, Italy; 6 National Research Council, Institute of Sciences of Food Production (CNR-ISPA), 70126 Bari, Italy; isabella.dantuono@cnr.it

**Keywords:** breadmaking, FODMAPs, fructans, irritable bowel syndrome, lactic acid bacteria, wheat flour

## Abstract

Irritable bowel syndrome (IBS) symptoms are often triggered by the consumption of fermentable oligosaccharides, disaccharides, monosaccharides, and polyols (FODMAPs), which are widely found in cereal-based foods, dairy products, fruits, legumes, and vegetables. Among these, cereal-based products are one of the main dietary sources of fructans. Consequently, low-FODMAP dietary strategies have gained increasing attention as an effective approach for managing IBS symptoms. This study adopted an integrated strategy based on the parallel selection of low-FODMAP flours and the screening of lactic acid bacteria (LAB) and yeasts, with the aim of identifying the best-performing flour and microbial starter to support the of low-FODMAP bread formulation. Sixteen soft and durum wheat flours were characterized for fructan content, chemical, physical, and rheological properties, revealing marked variability among the samples and highlighting the importance of raw material selection. Together, 150 LAB and 7 commercial yeasts were evaluated for their ability to modulate fructans, fructose, glucose and mannitol during 24 h model-dough fermentation. Most strains promoted substantial reduction in FODMAP levels, with variability depending on microorganisms, inoculum concentration, and fermentation time. Overall, the findings of this study provide a foundation for subsequent investigations focused on the development and characterization of low-FODMAP bread using the selected flour-starter combinations.

## 1. Introduction

FODMAPs (Fermentable Oligo-, Di-, Mono-saccharides and Polyols) are a group of short-chain carbohydrates that may induce gastrointestinal symptoms in individuals with irritable bowel syndrome (IBS), due to limited absorption in the small intestine and rapid fermentation by the gut microbiota [1,2,3]. IBS is a chronic and debilitating gastrointestinal disorder that currently affects 9–23% of the world’s population and is associated with alterations in gut–brain interactions, visceral hypersensitivity, and an impaired quality of life [4,5,6,7]. Among the dietary strategies available, the low-FODMAP diet is currently recognized as an effective approach to managing symptoms [8,9,10].

The international definition and classification of low-FODMAP foods is based on standardized compositional criteria derived from clinical challenge studies and analytical quantification methods [11,12]. These criteria establish threshold values for individual FODMAP groups and emphasize the importance of portion sizes in determining dietary suitability. In particular, a fructan content below approximately 0.3 g per serving is generally considered tolerable for most IBS patients [12,13]. However, long-term dietary restriction may present practical and nutritional challenges, including the potential impact on dietary variety and gut microbiota composition. Therefore, technological strategies aimed at directly reducing the FODMAP content of staple foods, such as bread, may represent a more sustainable and complementary approach.

FODMAPs include galacto-oligosaccharides (GOS), fructans and fructo-oligosaccharides (FOS), as well as lactose, fructose, sorbitol, xylitol and mannitol. Among these compounds, fructans represent the main FODMAP fraction present in cereal-based products, particularly those derived from wheat [8,14,15,16]. Considering that wheat bread constitutes a major source of fructan in Western diets [15,17], even moderate variations in flour composition may substantially affect overall FODMAP intake. The present study mainly focuses on fructans, which constitute the predominant fraction of FODMAPs in wheat. This choice is related to the preliminary nature of the investigation, aimed at screening the raw materials selected for the development of a low-FODMAP bread formulation. The remaining FODMAP components will be further investigated and quantified in the final product in future studies.

The fructan content of wheat varies greatly depending on wheat species (soft vs. durum wheat), genotype, environmental conditions, and milling degree [18,19,20]. Moreover, fructans are unevenly distributed within the grain, with higher concentrations generally found in the outer layers due to their accumulation in bran tissues [21,22]. Consequently, flour extraction rate and milling practices can substantially affect the final fructan content of wheat-based products. Reported fructan levels in cereal flours and processed bakery products range from approximately 0.7 to over 2.5 g/100 g dry weight, depending on both raw material characteristics and technological processes involved [2,23,24]. In addition to total concentration, the degree of polymerisation (DP) of wheat fructans may also influence their fermentability and potential physiological impact, further contributing to qualitative variability among flours [19,22]. These observations highlight the importance of flour selection as a preliminary step in the development of cereal-based products with reduced FODMAP content.

From a technological standpoint, fructans influence dough rheology, water absorption, gas retention, and the overall bread structure [18,25]. Therefore, their reduction may compromise technological performance if not carefully managed. Moreover, conventional bakery quality parameters do not reliably predict fructan content, meaning direct analytical assessment is essential when selecting raw materials for low-FODMAP applications.

The natural variability documented among wheat flours also suggests the potential to select raw materials with an intrinsically lower FODMAP content. This is a preliminary step in the development of cereal-based products for sensitive consumers, which is often overlooked.

In recent years, considerable efforts have been devoted to reducing FODMAP levels in wheat-based products through enzymatic treatments, ingredient reformulation, and fermentation-based strategies, particularly sourdough processes employing selected lactic acid bacteria (LAB) and yeasts [14,26,27,28,29]. Among these strategies LAB have shown promising potential for fructan degradation due to their strain-dependent enzymatic activities [30,31,32]. However, fructan hydrolysis does not necessarily correspond to a proportional decrease in total FODMAP content, since the released fructose may be further metabolized and converted into secondary fermentable compounds such as mannitol, which is classified as a FODMAP [8,15,33]. Consequently, effective low-FODMAP strategies should consider the overall carbohydrate balance generated during fermentation, rather than focusing exclusively on fructan breakdown [34].

Within this framework, the present study aimed to comprehensively characterize several soft and durum wheat flours intended for bread production by assessing their chemical composition, physical and rheological properties, and total fructan content, with the objective of evaluating their suitability for low-FODMAP applications. In parallel, the capacity of selected LAB and yeasts to modulate fructans and other FODMAP-related carbohydrates during wheat dough fermentation was evaluated on a strain-specific basis. By integrating flour selection with microbial screening, this study seeks to identify the most promising flour-starter combinations for developing fermented breads with reduced FODMAP content, improved tolerability for individuals with IBS, and preserved technological quality.

## 2. Materials and Methods

### 2.1. Flour Samples

A total of sixteen commercial flour samples, supplied by different producers, were analyzed, including nine soft wheat flours (code 1–9) and seven durum wheat flours (code 10–16). All samples were stored under dry conditions at room temperature until analysis.

### 2.2. Analytical Characterization of Soft and Durum Wheat Flours

The flour samples were characterized for chemical composition (protein, ash and moisture content), water activity (aw), rheological properties (Farinograph and Alveograph parameters), and fructan content. The analytical procedures used for each determination are described below.

Moisture content was determined by oven-drying according to standard methods (GU No. 285, 15-11-1967 [35]; ICC 110/1 [36]; UNI EN ISO 712 [37]). Ash content was determined by incineration in a muffle furnace at 550 ± 10 °C, following ICC 104/1 [38], AACC 08-12 [39] and UNI EN ISO 2171 [40] methods. Protein content, yellow index and gluten index were determined by near-infrared spectroscopy using a Foss XDS instrument. Water activity (aw) was measured using a Novasina Labmaster analyzer (Novasina AG, Lachen, Switzerland).

Dough water absorption and stability were measured using a Brabender Farinograph (Brabender GmbH & Co. KG, Duisburg, Germany), according to ICC standard methods. Alveographic parameters, including dough strength (W) and the P/L ratio, were determined using an AlveoPC Chopin alveograph (Chopin Technologies, Villeneuve-la-Garenne, France).

Total fructan content in flours was determined using an enzymatic assay kit (K-FRUC, Megazyme International, Bray, Ireland), according to the manufacturer’s instructions. Results were expressed as g fructans per 100 g of flour.

### 2.3. Lactic Acid Bacteria Strains

A total of 150 LAB strains, belonging to the microbial collection of the Institute of Food Sciences, National Research Council (ISA-CNR, Avellino, Italy) and listed in Supplementary Table S1, were evaluated in this study.

#### 2.3.1. Lactic Acid Bacteria Fermentation Experiments

##### Dough Preparation and LAB Inoculum

Lactic acid bacteria were evaluated for their ability to reduce fructan content during model wheat dough fermentation. Prior to inoculation, LAB strains were individually cultivated in De Man, Rogosa and Sharpe (MRS) broth (Oxoid, Milan, Italy) until the late exponential growth phase (approximately 16 h). Cells were harvested by centrifugation (10,000× *g* for 10 min at 4 °C), washed twice with 50 mM phosphate buffer (pH 7.0), and resuspended in the same buffer. The optical density of the cell suspensions was adjusted to achieve an initial bacterial concentration of approximately 7 log CFU/g of dough. Model doughs were prepared using 100 g of commercial type 0 wheat flour, sterile water (approximately 60 mL), NaCl (2.0% *w*/*w*) and were inoculated with the respective LAB suspensions. A non-inoculated dough was used as the control. All ingredients were mixed to obtain homogeneous doughs, which were incubated at 28 °C for 24 h. Fermentation was monitored at three time points: 0, 6 and 24 h of fermentation. At each time point, pH, total titratable acidity (TTA), LAB and yeast counts (Log CFU/g), and fructan content were determined.

##### pH and TTA Determination

The pH was measured using a BASIC 20 pH meter (Crison Instruments, Alella, Spain) on 10 g of dough that had been suspended in 90 mL of bidistilled water and homogenized for 2 min using a Stomacher (BagMixer 400, Interscience, Saint-Nom-la-Bretèche, France). The same suspension was used to determine TTA, which was expressed as the volume (mL) of 0.1 M NaOH required to reach pH 8.3. All measurements were performed in triplicate.

##### Microbiological Analyses

Microbiological analyses were carried out by homogenizing 10 g of each sample with 90 mL of sterile peptone water (1 g L^−1^ bacteriological peptone) for 2 min using a Stomacher (BagMixer 400, Interscience, Saint-Nom-la-Bretèche, France). Serial decimal dilutions were prepared, and LAB were enumerated on MRS agar (Oxoid, Milan, Italy), supplemented with cycloheximide (250 mg L^−1^), after incubation at 28 °C for 48–72 h. Yeasts were counted on YPD agar (10 g L^−1^ yeast extract, 20 g L^−1^ peptone, 20 g L^−1^ glucose, and 20 g L^−1^ agar) after incubation at 25 °C for 72–96 h. Results were expressed as Log CFU/g and reported as the mean ± standard deviation of three independent determinations.

##### Fructan Determination

Total fructan content was determined using an enzymatic assay kit for fructans (K-FRUC; Megazyme International, Bray, Co. Wicklow, Ireland), according to the manufacturer’s instructions. The test principle consists of a preliminary hydrolysis that involves the removal of starch and sucrose with specific sucrase enzymes; the conversion of glucose, fructose, and sucrose-free sugar alcohols by sodium borohydride (fructan remains unchanged); hydrolysis of fructans in glucose and fructose by exoinulinase, endoinulinase, and endoglucanase; and a final quantification of the released reducing sugars (PAHBAH method).

#### 2.3.2. Assessment of Selected Lactic Acid Bacteria Strains in Model Dough Fermentations and Carbohydrate Profiling

After the preliminary screening described in Section 2.3.1, new dough fermentation experiments were conducted to assess whether fructan degradation resulted in an increase in other FODMAP compounds. During fermentation, the levels of fructose, mannitol, and glucose were also monitored. Dough preparation and LAB inoculation were performed as described in Section Dough Preparation and LAB Inoculum. The level of pH, TTA, LAB and yeast counts, and fructan content were evaluated as described in Section pH and TTA Determination, Section Microbiological Analyses, and Section Fructan Determination, respectively. Mannitol, fructose, and glucose were quantified using enzymatic mannitol assay kits (K-MANOL), and fructose–glucose (K-FRUGL) (Megazyme International, Bray, Co. Wicklow, Ireland), according to the manufacturer’s instructions.

### 2.4. Assessment of Commercial Bakery Yeasts Able to Reduce Fructan Content in Model Dough Fermentation

Seven commercial bakery’s yeasts (including compressed and dried formulations) were evaluated in model doughs to assess their ability to reduce fructan content during dough fermentation.

The amount of each yeast preparation was applied according to the specific instructions reported on the product labels, as these products differ in formulation, recommended dosage, cell concentration, and cell viability. The exact formulation details and the actual yeast counts determined after dough inoculation are presented together with the corresponding results to facilitate data interpretation. However, to allow a comparable evaluation of the leavening performance among the different commercial yeasts, the fermentation endpoint was defined based on dough volume development. Fermentation was stopped when dough volume approximately doubled, which represents a commonly adopted practical criterion in bread-making processes. The fructan content, pH and yeast counts were measured at time zero (immediately after inoculation) and at the end of fermentation, defined as the point at which the dough volume had doubled, as described in Section pH and TTA Determination, Section Microbiological Analyses, and Section Fructan Determination, respectively.

### 2.5. Statistical Analysis

All analyses were performed in triplicate, and the results are expressed as the mean values ± standard deviation. Statistical analysis was carried out using XLSTAT software (version 2020). Differences among samples were evaluated by one-way analysis of variance (ANOVA), followed by Tukey’s post hoc test for multiple comparisons. Differences were considered statistically significant at *p* < 0.05.

Before performing PCA, the variables were mean-centered and standardized by unit variance scaling to remove the influence of different measurement scales among variables. Correlation matrix was performed using Pearson coefficients.

## 3. Results and Discussion

### 3.1. Flour Characterization

The characterization of the soft and durum wheat flours used in the study was carried out to evaluate their technological performance and fructan content. A summary of the main physicochemical and rheological parameters, reported as mean values for soft and durum wheat flour groups, is presented to facilitate a general comparison between the two categories. Results are shown in Table 1.

The comparative analysis of soft and durum wheat flours highlighted significant differences in several technological (W, P/L, water absorption) and compositional parameters (ash, protein content), while no significant differences were observed in moisture content, fructan content, and dough stability.

Durum wheat flours showed significantly higher ash content (1.35% vs. 0.63%, *p* = 0.031) and protein content (13.47% vs. 11.16% d.m., *p* = 0.007) compared to soft wheat flours. The higher ash levels are consistent with the greater extraction rate and higher bran particle inclusion typically associated with durum wheat milling. This may also contribute to the increased mineral content observed.

Significant differences were also observed for the yellow index, with durum wheat flours showing markedly higher values than soft wheat flours (*p* < 0.001), reflecting their higher carotenoid pigment content [41]. Water activity did not differ significantly between the two flour types.

**Figure 1:**
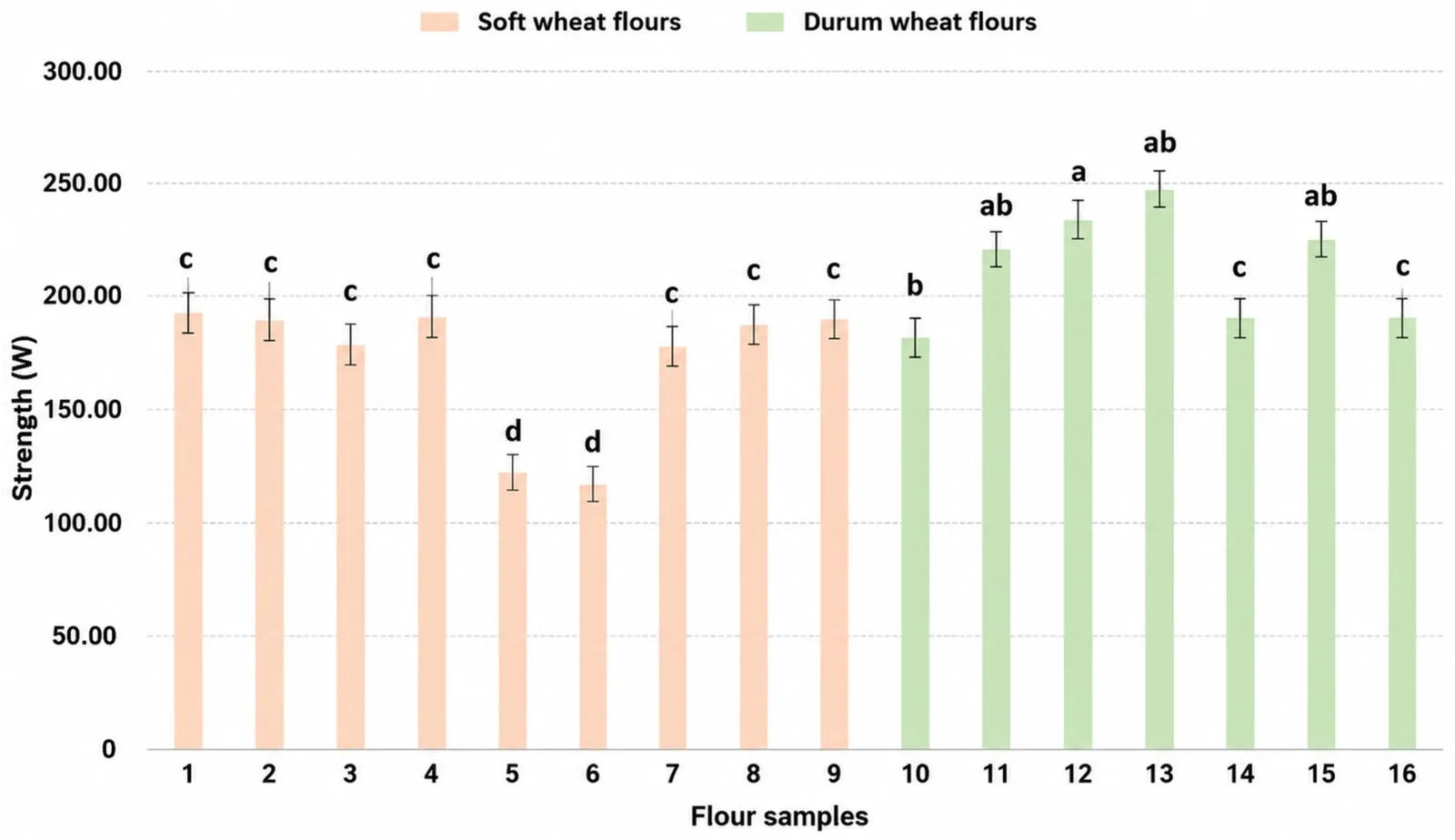
Alveographic dough strength (W) of the analyzed flour samples. Different letters (a, b, c, d) indicate significant differences between samples (*p* < 0.05).

The higher protein content resulted in significantly stronger gluten properties, as also reflected by the higher W values, a key rheological descriptor for baking performance, observed in durum flours compared to soft flours (212 vs. 171, *p* = 0.012). Moreover, substantial variability in the W parameter was observed among the individual flour samples, ranging from 118 to 248. This variability is further highlighted in Figure 1 which clearly illustrates the differences across the 16 flour samples analyzed.

In particular, certain soft wheat samples (5, 6) showed reduced gluten strength (W < 130), whereas several durum wheat samples exceeded 220 (samples 11, 12, 13, 15), indicating strong and highly tenacious dough properties.

The broad distribution of W values confirms the heterogeneity of technological quality among the examined flours. The observed variability among samples may be attributed to the analysis of different commercial batches of the same flour type rather than replicates from a single batch.

The alveographic configuration differed markedly between flour types. Durum wheat flours exhibited significantly higher P/L ratios (2.26 vs. 1.01, *p* = 0.001), reflecting a more tenacious and less extensible gluten network. This rheological profile may influence gas retention, dough machinability, and fermentation dynamics, potentially affecting microbial activity during prolonged fermentation.

Water absorption was also significantly higher in durum wheat flours (64.72% vs. 55.22%, *p* = 0.005), likely due to their higher protein and ash contents, which increase water-binding capacity [42]. Since hydration level strongly influences enzymatic activity and microbial growth, these differences could indirectly affect fructan hydrolysis during fermentation.

As reported in Table 1, moisture content did not differ significantly between soft and durum wheat flours, suggesting comparable storage and processing conditions among the analyzed samples.

Similarly, no significant differences in farinograph stability were observed between the two flour types, despite the higher gluten strength typically associated with durum wheat [43]. This finding suggests that dough mixing tolerance is not directly correlated with alveographic strength.

**Figure 2:**
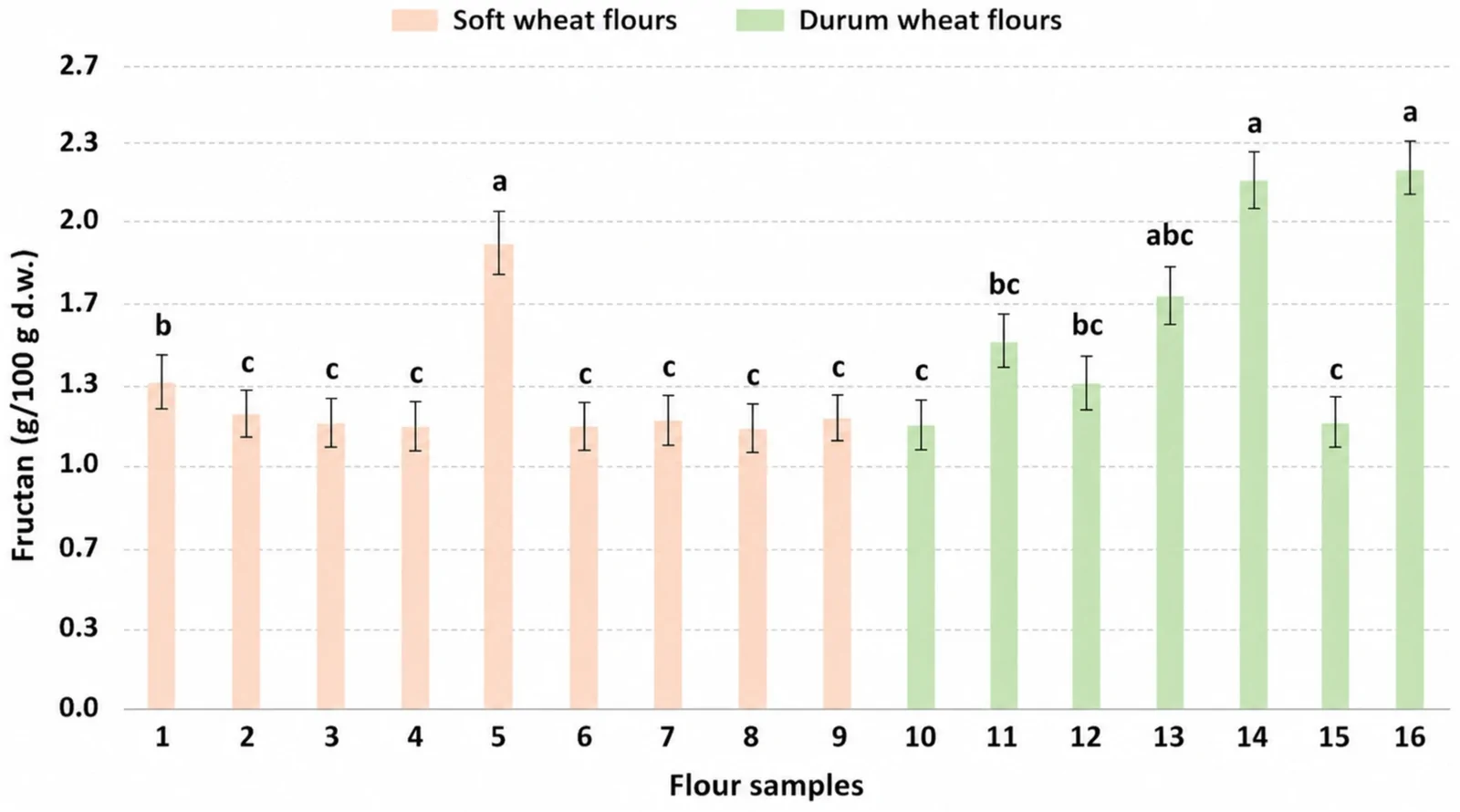
Total fructan content of the analyzed flour samples. Different letters (a–c) indicate significant differences between samples (*p* < 0.05).

In addition, no significant difference was observed in total fructan content between soft and durum wheat flours (1.26 vs. 1.65 g/100 g d.m., n.s.). However, although no significant differences were observed between soft and durum wheat flours, significant intra-group variability was detected within both categories. Total fructan content ranged from 0.93 to 2.14 g/100 g dry matter among the analyzed flour samples (Figure 2), confirming the wide variability reported in the literature for wheat flours [21,44,45].

In particular, some samples exhibited fructan levels more than twofold higher than others, highlighting substantial variability among individual flours. This suggests that differences in fructan content cannot be explained solely by wheat type (durum or soft wheat), as the variability within each group may exceed the differences between them. From a low-FODMAP perspective, this result highlights the importance of analytical screening of individual flours, rather than selecting them based only on their botanical classification.

**Figure 3:**
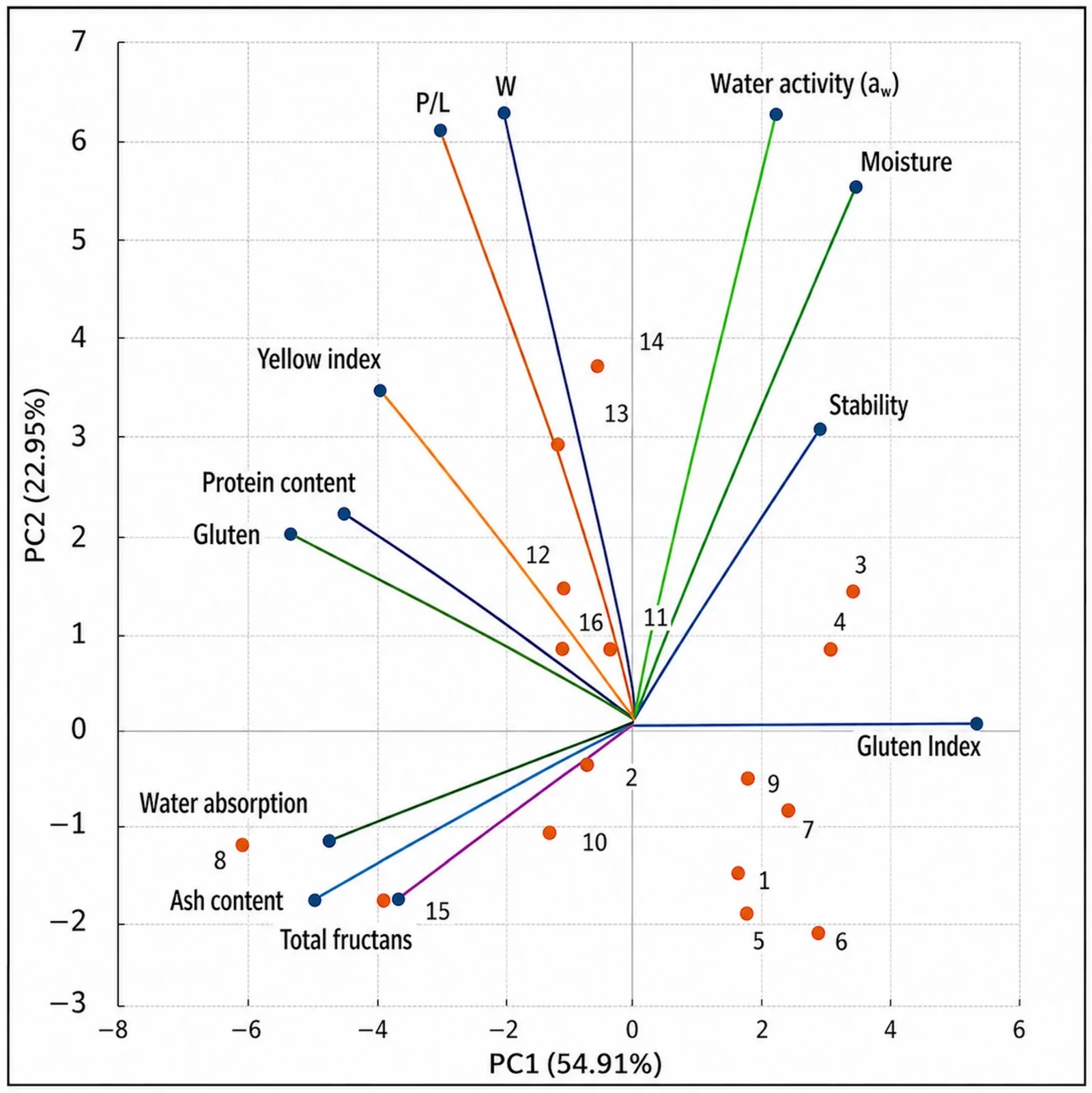
Principal Component Analysis (PCA) of flour samples based on chemical, physical and rheological parameters. Score plot showing the distribution of soft wheat and durum wheat flours with 95% confidence ellipses. Loading plot indicating the contribution of the variables to PC1 and PC2. Note: 1–9: soft wheat flours; 10–16: durum wheat flours.

To obtain a comprehensive overview of the relationships between compositional and rheological parameters, and to better discriminate flour samples, Principal Component Analysis (PCA) was performed (Figure 3).

The first two PCs globally explained 77.86% of the total variability. The score plot showed a clear separation of the samples, mainly along the first principal component (54.91%) and according to flour type. Soft wheat flours were primarily associated with higher values of water activity, moisture content, water absorption, dough stability, and gluten index, whereas durum wheat flours were positioned on the opposite side of the component.

The loading plot confirmed that the discrimination among the samples was driven by a combination of chemical and rheological parameters, highlighting the relevance of flour type in defining technological properties.

The sample distribution indicated a partial separation between soft and durum wheat flours along PC1, although overlap was observed, reinforcing the concept that the variability within flour types may exceed inter-type differences. Although total fructan content was generally higher in durum wheat flours than in soft wheat flours, no statistically significant differences were detected between the two groups, in agreement with previous literature [19].

A significant positive correlation was observed between ash content and total fructan content (R = 0.797; *p* < 0.05), supporting literature evidence that fructans are mainly concentrated in the bran fraction of wheat kernels [21,46]. Furthermore, water absorption was positively correlated with both ash content (R = 0.954; *p* < 0.05) and total fructan content (R = 0.776; *p* < 0.05), in line with findings reported by Pejcz et al. [14].

Based on the combined evaluation of fructan content and rheological performance, six soft wheat flour samples (No. 2, 3, 4, 7, 8 and 9) and two durum wheat flour samples (No. 10 and 15) were selected as the most suitable candidates for further biotechnological trials.

Overall, the integrated assessment of compositional and rheological parameters demonstrates that flours with comparable technological strength may differ substantially in FODMAP levels. This variability reinforces the need for a multidimensional selection strategy when designing wheat-based products with reduced fermentable carbohydrate content.

The observed technological differences between flour types may also influence microbial behavior during fermentation, potentially modulating the kinetics of fructan degradation and secondary FODMAP formation.

### 3.2. Selection of LAB Able to Reduce Fructans in Model-Dough Fermentation

#### 3.2.1. pH, Total Titratable Acidity (TTA), LAB Count and Fructan Evolution During 24 h Fermentation of Model-Dough

**Figure 4:**
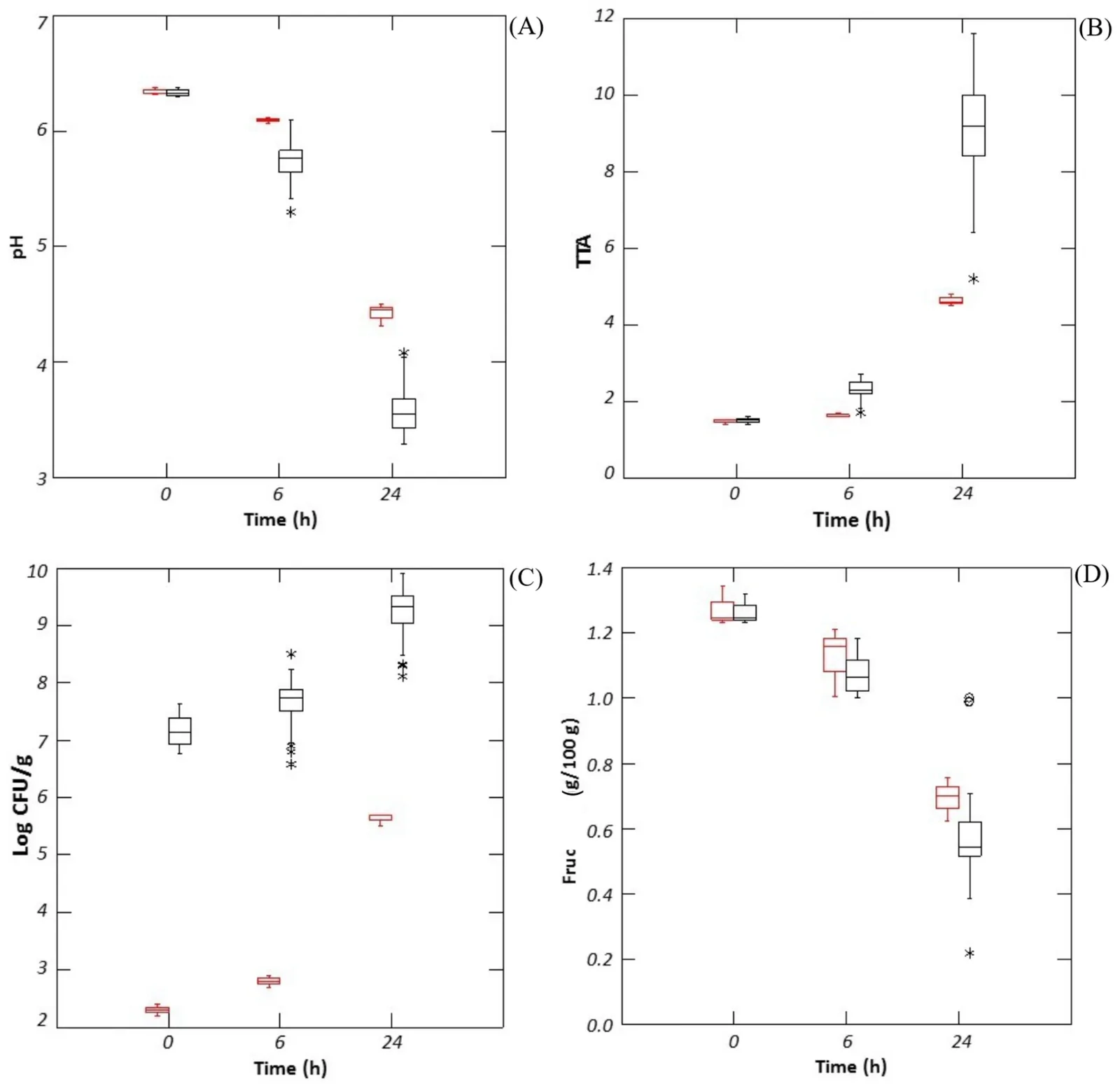
Boxplot distribution of pH (**A**), TTA (**B**), LAB concentration (**C**), Fructan content (**D**) values measured at 0, 6, and 24 h during fermentation of model doughs inoculated with 150 lactic acid bacteria strains. Black boxplots represent inoculated dough samples, whereas red boxplots correspond to control doughs without inoculum. Outliers and far outliers are markedwith asterisk (*) and circle (o).

A total of 150 strains of lactic acid bacteria were evaluated for their ability to degrade fructans during 24-h fermentation of model dough. The results for pH, TTA, microbial cell density (Log CFU/g), and fructan content at 0, 6, and 24 h of fermentation are reported in Figure 4.

Boxplot A shows the distribution of pH values. At time 0, the pH values were highly homogeneous, with a value around pH ~ 6.3, indicating that all fermentations started from comparable initial conditions. After 6 h of fermentation, a slight decrease in pH was observed in the inoculated samples, with median values around 5.7–5.8. After 24 h, a marked drop in pH was evident, with median values around 3.5–3.6, indicating intense acidification of the dough. The distribution is broader compared to time zero, highlighting significant differences in acidification kinetics depending on the strain. Control dough samples without bacterial inoculation were also monitored over time. The initial pH of these samples was similar (~6.3), decreasing slightly to around 6.1 after 6 h and reaching approximately pH 4.5 after 24 h. This moderate acidification indicates limited microbial activity, likely due to the presence of endogenous microorganisms naturally associated with flour and dough. Overall, the results demonstrate progressive dough acidification during fermentation driven by LAB metabolic activity, with increasing variability over time reflecting strain-dependent differences in acidification performance.

Boxplot B shows the distribution of TTA values. At time 0, TTA values were low and highly uniform, with median values of around 1.2–1.3 mL. The narrow distribution indicates that all doughs started from comparable baseline conditions, as was also observed for the pH values. After 6 h of fermentation, a moderate increase in TTA was observed in the inoculated samples, with median values of around 2.1–2.3 mL, reflecting the onset of organic acid production by LAB during carbohydrate metabolism. After 24 h, a substantial increase in TTA was detected, with median values approaching 9–10 mL, indicating strong accumulation of organic acids in the dough. The wider distribution observed at this time point highlights considerable variability among the strains in their acidification capacity. Control dough samples without bacterial inoculation showed TTA values similar to those at time 0 and after 6 h of fermentation. However, after 24 h TTA values increased to approximately 5 mL, indicating a moderate acidification driven by endogenous microbiota naturally present in the flour and dough. The gradual increase in TTA over fermentation is consistent with the pH decrease observed during fermentation, confirming the strong relationship between acid production and dough acidification. Similar trends have been widely reported in sourdough fermentations, where LAB activity leads to a gradual accumulation of organic acids [47,48,49,50]. The greater variability observed after 24 h likely reflects strain-dependent metabolic differences, including variations in carbohydrate utilization pathways, growth kinetics, and the balance between homo- and hetero-fermentative metabolism, which represent key technological traits in sourdough fermentation and starter culture selection [51,52,53].

Boxplot C shows the distribution of microbial load (Log CFU/g). At time 0, microbial counts ranged approximately between 6.8 and 7.5 Log CFU/g, with a median of around 7.0 Log CFU/g, reflecting the initial inoculum level and the relatively homogeneous starting conditions. After 6 h of fermentation, an increase in microbial counts was observed, with median values of around 7.6–7.7 Log CFU/g, suggesting active growth and adaptation of LAB strains to the dough environment. However, the distribution was slightly wider compared to time 0, suggesting differences in growth kinetics. After 24 h, counts reached about 9.1–9.2 Log CFU/g, values typical of mature sourdough, confirming successful proliferation of most strains. The broader distribution reflects variability in growth performance depending on the strain, which may be influenced by differences in carbohydrate metabolism, tolerance to acidic conditions, and adaptation to cereal substrates. Control dough samples showed an initial LAB count of about 2.3 Log CFU/g, increasing slightly to around 3 Log CFU/g after 6 h and reaching approximately 6 Log CFU/g after 24 h. This increase, although at lower levels than those observed in the inoculated samples, suggests the growth of endogenous microorganisms naturally present in the flour, which contribute to the establishment of the typical sourdough ecosystem [54,55,56]. Overall, these results confirm that most of the tested strains were able to grow effectively in the model dough system, reaching high cell densities after 24 h of fermentation, albeit with noticeable strain-dependent differences. The results of the yeast count analysis were not reported, as all values were consistently below 2 Log CFU/g; the analysis was conducted solely to assess the presence of potential contamination.

Boxplot D shows the fructan concentrations in model doughs during fermentation. At the start of the experiment, the fructan concentration was uniform across all doughs, with a median value of approximately 1.25 g/100 g. After 6 h of fermentation, a very low reduction in fructan levels was observed, with the median dropping to approximately 1.05 g/100 g. The most significant findings occurred after 24 h of fermentation where the overall median fructan concentration decreased to approximately 0.5 g/100 g, representing a total reduction of over 50% compared to T0. Furthermore, the graph indicates high variability in degradation efficiency among the strains.

Even the control dough samples without bacterial inoculation showed a gradual decrease in fructan concentration during fermentation. In these samples, fructan levels decreased from an initial value of around 1.25 g/100 g to approximately 1.1 g/100 g after 6 h of fermentation. After 24 h, the control dough had fructan concentrations of approximately 0.7 g/100 g.

This reduction suggests that, regardless of the LAB strains inoculated, the degradation of fructans is also strongly dependent on the acidification of the dough.

Notably, a significant drop in pH was observed across all doughs, peaking after 24 h of fermentation. This acidification probably favored fructan reduction through two synergistic mechanisms: first, by providing the optimal acidic environment (typically pH 4.0–5.0) for the activation of endogenous cereal invertases naturally present in the flour [57]; and second, through direct acid-catalyzed hydrolysis of the fructan chains [58]. Consequently, while strain-specific activity remains the primary driver of diversity, the general acidification of the matrix acts as a fundamental catalyst for the overall degradation process.

#### 3.2.2. Advance Selection of LAB Strains

Based on the results obtained in the previous experiment, a subset of strains was selected based on their ability to achieve the greatest reduction in fructan content, using a threshold of 0.5 g/100 g. Consequently, twenty-five strains capable of reducing fructan concentrations below this threshold were selected for further study.

These strains were then used in new 24 h fermentations of model doughs to verify if fructan degradation increased the concentration of the other FODMAP compounds as fructans, fructose and mannitol.

**Figure 5:**
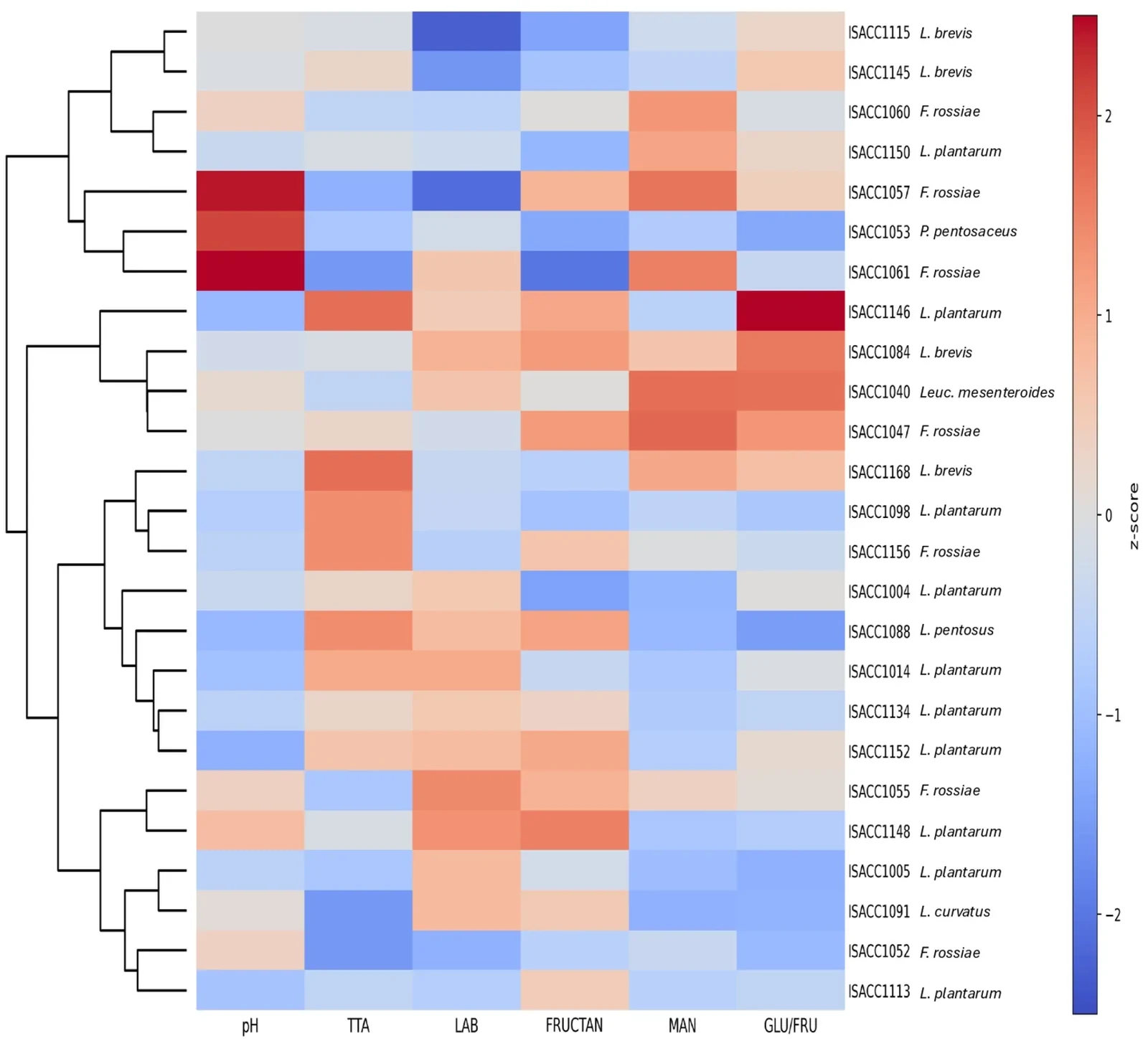
Matrix Hierarchical Cluster Analysis. Colors represent standardized values (z-scores), with red indicating higher values and blue indicating lower values. Rows correspond to strains, while columns represent the measured variables (pH, TTA, LAB, fructan, mannitol, and glucose/fructose ratio). Hierarchical clustering is shown by the dendrogram, grouping samples with similar profiles.

Results are summarized in Figure 5, which presents the hierarchical clustering heatmap of the strains based on pH, total titratable acidity (TTA), viable cell counts, fructan contents, glucose/fructose ratio and mannitol production. The heatmap revealed distinct phenotypic profiles, with strains exhibiting similar metabolic and technological characteristics clustering together, indicating consistent phenotypic behavior across the evaluated parameters. Moreover, the observed grouping highlights the phenotypic heterogeneity among the strains, reflecting differences in their metabolic performance and technological potential.

In detail, all of the tested strains showed good adaptation to the dough environment. Final pH values ranged from 3.3 to 4.0, indicating effective acidification across all samples. TTA values (8.0–11.6 mL) were consistent with typical sourdough fermentation while LAB counts (~8.5–9.7 Log CFU/g) confirmed robust microbial growth in all trials. Overall, no strain showed technological limitations in terms of fermentation performance. Fructan levels varied moderately across the samples (≈0.38–0.60 g/100 g), indicating that strain selection had a limited yet noticeable effect on fructan reduction. The lowest fructan contents were observed in *Furfurilactobacillus rossiae* 1061 (0.3847 g/100 g), *Lactiplantibacillus plantarum* 1004 (0.4020 g/100 g), *Pediococcus pentosaceus* 1053 (0.4060 g/100 g). However, these differences were relatively small, suggesting that fructan degradation was not the primary discriminating factor among strains.

Clear strain-dependent differences emerged for mannitol accumulation. High mannitol levels were associated with heterofermentative species such as *Levilactobacillus brevis* 1168 (up to 0.376 g/100 g), *F. rossiae* 1047 (up to 0.495 g/100 g), and *Leuconostoc mesenteroides* 1040 (0.483 g/100 g). This observation can be explained by the metabolism of heterofermentative lactic acid bacteria. As reported by Gänzle and Gobbetti [59], most heterofermentative LAB reduce fructose to mannitol as a strategy for cofactor regeneration, using fructose as an external electron acceptor, and this conversion is particularly favored under acidic conditions. This mechanism is mediated by NADH-dependent mannitol dehydrogenase, which regenerates NAD+ while reducing fructose to mannitol. Consistent with this general metabolic pathway, other authors identified *Levilactobacillus brevis* [60] and *L. mesenteroides* [61] as efficient mannitol-producing heterofermentative LAB, demonstrating their ability to convert fructose quantitatively into mannitol through this redox-balancing mechanism. Therefore, the high mannitol accumulation observed in the present study in doughs containing heterofermentative lactic acid bacteria is likely explained by the combined effect of increased fructose availability, resulting from fructan hydrolysis, and the intrinsic heterofermentative metabolism of this species.

In contrast, very low mannitol concentrations were found in *Latilactobacillus curvatus* 1091 (0.016 g/100 g), *L. plantarum* 1004 (0.030 g/100 g), *L. pentosus* 1088 (0.031 g/100 g), *L. plantarum* 1005 (0.048 g/100 g), *L. plantarum* 1014 (0.077 g/100 g). These results confirm that mannitol production is strongly linked to heterofermentative metabolism, where fructose acts as an electron acceptor [62].

Accordingly, mannitol production was identified as a key discriminating factor among strains, and the selection was initially restricted to those exhibiting low mannitol production.

Within this group, a further selection criterion based on the GLU/FRU ratio was applied, as this is an indicator of residual fructose levels. The GLU/FRU ratio showed wide variability (0.5–6.7). Among the strains showing low mannitol accumulation, selection was further refined based on the GLU/FRU ratio, with preference given to strains with higher values, which are indicative of lower residual fructose concentrations.

According to these criteria, selected strains of *L. plantarum*, particularly strains 1004 and 1014, emerged as the most promising candidates, due to their combination of low mannitol accumulation, acceptable fructan reduction, and balanced GLU/FRU ratio.

The superior performance of *Lactiplantibacillus plantarum* observed in the present study is consistent with previous findings reported by Marín-Sanz et al. [23], who identified selected homofermentative *L. plantarum* strains as promising starter cultures for the production of low-FODMAP whole-wheat bread. In their study, the ability to reduce fructan content was shown to be strongly strain-dependent, highlighting the importance of careful starter selection.

The high efficiency of *L. plantarum* may be attributed to its remarkable metabolic versatility and its ability to rapidly acidify the dough environment, creating favorable conditions for carbohydrate metabolism and fructan utilization. In addition, *L. plantarum* is recognized for its broad repertoire of carbohydrate-active enzymes, which may contribute to the degradation of fructans either directly or through synergistic interactions with baker’s yeast during fermentation. Although the exact mechanisms were not investigated in this study, the observed results further support the suitability of selected *L. plantarum* strains as key components of mixed starter cultures for developing low-FODMAP bakery products.

Therefore, *L. plantarum* 1004 (named later LA1) and *L. plantarum* 1014 (LA2) were considered suitable candidates for low-FODMAP sourdough production, especially considering their stable technological performance and were used in the subsequent baking trials. Although strain selection was based on the fermentation endpoint (24 h), monitoring intermediate fermentation stages would provide valuable information on the kinetics of fructan degradation and carbohydrate metabolism. Future work will therefore include time-course analyses to characterize the degradation profiles of the selected strains throughout fermentation.

### 3.3. Selection of Commercial Bakery Yeasts Able to Reduce Fructans in Model-Dough Fermentation

Seven commercial bakery’s yeasts were screened for their ability to degrade fructans in model dough, with the aim of identifying the most effective ones under short fermentation conditions. The results of the fructan content, pH, and yeasts counts during fermentation are reported in Table 2.

At time zero, the doughs showed an initial fructan content of approximately 1.2 g/100 g, a yeast inoculum of about 8 Log CFU/g for compressed fresh bakery’s yeast, and values between 5 and 7 Log CFU/g for active and dried yeasts, with an initial pH of around 6.2. After fermentation, all samples exhibited a substantial reduction in fructan content, indicating that the yeasts were able to metabolize fructans during leavening.

The doughs inoculated with fresh compressed yeast (samples A, B, C, D) showed similar leavening times (3 h and 30 min) and yeast counts at the end of fermentation (around 8 Log CFU/g). Fructan levels in these samples ranged from 0.2056 to 0.2987 g/100 g, corresponding to a strong decrease compared to the initial value. Among them, sample A showed the lowest fructan concentration (0.2056 g/100 g).

Active dry yeast samples (F and G) required longer leavening times (4 h 30 min and 7 h 30 min, respectively). Sample G showed a relatively low fructan content (0.2078 g/100 g), comparable to sample A, but had a markedly lower yeast count (5 Log CFU/g), suggesting reduced yeast viability or metabolic activity at the end of fermentation.

The dried yeast sample (H) exhibited the highest residual fructan content (0.5431 g/100 g), despite the long leavening time (7 h 30 min), indicating a lower capacity for fructan degradation. Regarding pH, all samples showed a slight decrease from an initial value of 6.2 to final values between 5.4 and 5.7, reflecting metabolic activity during fermentation.

Overall, considering the lowest residual fructan content, along with a high yeast population and a shorter fermentation time, the fresh compressed yeast A was identified as the best-performing yeast for subsequent bread-making trials involving co-fermentation with the selected LAB strains.

Subsequently, dough fermentation trials were carried out to evaluate fructan reduction using commercial yeasts (Y) and lactic acid bacteria (LA1 and LA2), previously selected as described in Section 3.2. A total of nine dough formulations were prepared (code A–I) as reported in Table 3. Three doughs (A, B and C) were fermented with commercial yeast alone, inoculated at three different concentrations (approximately 6, 7 and 8 Log CFU/g of dough. Two additional doughs were prepared using yeast (8 Log CFU/g of dough) and LA1 and LA2 individually inoculated at approximately 9 Log CFU/g. The remaining four dough (F, G, H, I) formulations combining the two strains of *L. plantarum* with the baker’s yeast, at different inoculation levels.

The experimental approach was intended to evaluate whether the simultaneous use of the two *L. plantarum* strains could provide complementary or synergistic effects, resulting in improved fructan degradation and overall fermentation performance compared with formulations containing a single LAB strain. Varying the inoculum ratios also allowed the influence of LAB concentration on the fermentation process to be assessed, with the aim of identifying the most effective mixed-starter formulation for low-FODMAP bread production.

The fermentation time was not fixed but depended on the microbial inoculum concentrations, which determined the dough leavening kinetics [29]. The first fermentation was considered complete when the dough volume doubled. The dough was then reshaped and subjected to a second proofing of approximately 30 min to restore its structure before baking. Overall fermentation times ranged approximately between 3.5 and 4.5 h depending on the inoculum level.

Dough acidification, microbial growth, and fructan levels were monitored in order to assess the conditions that ensured the best reduction in FODMAPs. The results of these analyses are reported in Table 3. Data regarding control sample (uninoculated) are not reported since the value of all the parameters resulted unchanged at the end of leavening.

The results reported in Table 3 show that the fermentation conditions strongly influenced dough acidification and fructan degradation. Doughs fermented only with yeast (Y) showed limited acidification, with final pH values ranging from 5.38 to 5.48 and relatively low TTA values (2.0–2.8 mL). Under these conditions, the fructan levels remained relatively higher (0.2193–0.3011 g/100 g).

In contrast, the addition of LAB in combination with yeast (Y) significantly increased dough acidification. Samples containing LAB showed lower pH values (3.84–4.77) and higher TTA values (3.2–5.6 mL), indicating a more intense fermentation process. This stronger acidification was associated with a greater reduction in fructans, confirming the important role of LAB metabolism in the degradation of FODMAP compounds during fermentation.

Among the formulations tested, the combination H, with an initial LAB and yeast concentration of about 9.7 Log CFU/g and 8.28 Log CFU/g, recorded a significative lowest fructan content (0.1747 g/100 g) after 3 h 40 min of leavening. This sample also showed the highest level of acidification (pH 3.84; TTA 5.60 mL). These findings suggest that the selected mixed-starter formulation provided the most effective fermentation performance under the experimental conditions adopted. Although the present study was not specifically designed to demonstrate synergistic interactions between the two *L. plantarum* strains, the superior performance of their combination may indicate complementary metabolic activities that enhanced fructan degradation and fermentation efficiency. A possible explanation is that the greater acidification promoted a more favorable environment for yeast invertase activity. A previously reported by Nilsson et al. [63] sourdough fermentation lowers pH towards the optimal range for yeast invertase, thereby enhancing its catalytic activity and leading increased fructan hydrolisis.

Therefore, the greater fructan degradation observed in our sample may not only reflect the combined metabolic activity of the selected LAB strains and yeast but also the indirect effect of acidification on enzyme efficiency, resulting in a more extensive breakdown of fructans.

Based on these results, an additional trial was carried out using a combination of yeasts (Y) (at about 8 Log CFU/g) and the LAB strains (LA1 and LA2) at two different concentrations (approximatively 8 and 9 Log CFU/g for each strain). The doughs were prepared and pH, TTA, yeast and LAB counts, glucose, fructose, mannitol and fructan content were measured at time zero and at the end of leavening (about 4 h and 30 min).

Results of the inoculated samples during fermentation are reported in Table 4**.** Data regarding control sample (uninoculated) are not reported since the value of all the parameters resulted unchanged at the end of leavening (3 h and 40 min).

At inoculation (time 0), the sample inoculated with the yeast starter (Y) exhibited low levels of LAB, deriving from flour contamination, and high yeast counts originating from the inoculum. At the end of leavening, the high yeast activity determined substantial sugar consumption, along with moderate acidification (pH 5.11; TTA 2.4 mL). The mixed starter samples inoculated with yeast and LAB at two different LAB concentration (8.38 ± 0.28 and 9.10 ± 0.11 Log CFU/g, respectively) showed significantly higher LAB counts, indicating successful inoculation with bacterial strains.

In contrast, the Y sample demonstrated strong yeast activity.

The mixed starter (LA1 + LA2 + Y) samples exhibited the most pronounced fermentation effects. LAB counts remained high or further increased, while glucose and fructose were more extensively depleted. Notably, mannitol was not detected in these samples. Fructan levels were significantly reduced compared to the control, indicating enhanced degradation.

Acidification was strongest in the mixed starter samples, with the lowest pH values (down to 4.29) and highest TTA values (up to 4.4 mL). This reflects the metabolic activity of LAB and their contribution to organic acid production.

Overall, the combined LAB and yeast starter (LA1 + LA2 + Y) resulted in the most efficient fermentation, characterized by high microbial activity, greater sugar consumption, fructan reduction, and increased acidification, which are desirable traits for improving dough fermentation performance and potential nutritional properties [64,65].

These results indicate that higher LAB concentrations combined with yeast fermentation improve fructan reduction, likely due to the activity of LAB involved in carbohydrate metabolism. Therefore, the mixed starter composed of LA1, LA2, and yeast at high LAB concentration was selected as the most effective condition for producing bread with a reduced fructan content and to be used in subsequent breadmaking trials targeting low-FODMAP bread.

## 4. Conclusions

In conclusion, this work represents a preliminary screening phase for the selection of suitable raw materials and microbial starters for the production of low-FODMAP bakery products.

This study provided a comprehensive characterization of soft and durum wheat flours intended for the production of low-FODMAP bread, highlighting a marked variability in chemical, physical and rheological properties, as well as in total fructan content. The correlations observed between ash content, fructans and rheological parameters, particularly water absorption, confirm the role of flour composition in influencing both FODMAP content and technological performance.

The subsequent fermentation trials demonstrated that changes in FODMAP levels were highly dependent by fermentation process. Selected mixed LAB and yeast cultures exhibited enhanced FODMAP degradation compared with individual strains, indicating potential synergistic interactions among microorganisms.

Based on the obtained results, one flour and one starter culture were identified as the most promising candidates and will be employed in subsequent breadmaking trials to assess their technological performance and effectiveness in reducing FODMAP content.

This integrated screening approach is a vital step towards formulating fermented bakery products with reduced FODMAP content while maintaining dough functionality and product quality. The present findings provide a promising basis for the development of low-FODMAP bread as a potential dietary option for individuals with IBS. Nevertheless, further investigations on the final product are warranted to achieve a more comprehensive characterization of its technological, nutritional, and clinical properties. Future studies will focus on bread production using the optimized fermentation conditions and will include texture profile analysis (TPA), sensory evaluation, and HS-GC-MS characterization of volatile compounds to assess the impact of fermentation strategy on the technological and organoleptic properties of the final product.

## Figures and Tables

**Table 1:** Chemical, physicochemical, rheological parameters and fructan content of soft and durum wheat flours (mean ± SD).

Parameter	Soft Wheat Flours (*n* = 9)	Durum Wheat Flours (*n* = 7)	*p*-Value
Moisture (%)	14.06 ^a^ ± 1.08	12.01 ^a^ ± 1.46	n.s.
Ash (%)	0.63 ^b^ ± 0.30	1.35 ^a^ ± 0.83	0.031
Water activity (a_w_)	0.595 ^a^ ± 0.053	0.607 ^a^ ± 0.065	n.s.
Yellow Index	15.88 ^b^ ± 2.59	23.59 ^a^ ± 2.33	<0.001
Protein (% d.m.)	11.16 ^b^ ± 1.29	13.47 ^a^ ± 1.66	0.007
W	171 ^b^ ± 29.71	212 ^a^ ± 25.95	0.012
P/L	1.01 ^b^ ± 0.35	2.26 ^a^ ± 0.80	0.001
Water absorption (%)	55.22 ^b^ ± 2.51	64.72 ^a^ ± 8.24	0.005
Fructans (g/100 g d.m.)	1.26 ^a^ ± 0.28	1.65 ^a^ ± 0.48	n.s.
Stability (min)	4.22 ^a^ ± 2.87	2.87 ^a^ ± 1.62	n.s.

Note: Values are expressed as mean ± standard deviation of soft wheat (*n* = 9) and durum wheat (*n* = 7) flour samples. Differences between flour types were evaluated by Student’s *t*-test (*p* < 0.05). Values within a row followed by different letters are significantly different. Abbreviations: W, dough strength; n.s., not significant.

**Table 2:** Characterization of doughs (recipe, yeast type) and analytical changes (pH, yeasts, fructans) at time zero (inoculation) and final proofing.

Code	Type of Starter	Recipe	Time Zero			End of Leavening			
			Yeast Count (Log CFU/g)	Fructan (g/100 g)	pH	Leavening Time	Yeast Count (Log CFU/g)	Fructan (g/100 g)	pH
A	Compressed fresh yeast	1 g yeast + 50 g flour + 1 g NaCl + 30 mL water	8.0 ± 0.11	1.253 ± 0.01	6.2 ± 0.11	3 h 30 min	7.9 ± 0.54	0.2056 ± 0.01	5.59 ± 0.37
B	Compressed fresh yeast	1 g yeast + 50 g flour + 1 g NaCl + 30 mL water	8.1 ± 0.07	1.253 ± 0.01	6.2 ± 0.11	3 h 30 min	8.0 ± 0.18	0.2754 ± 0.03	5.42 ± 0.45
C	Compressed fresh yeast	1 g yeast + 50 g flour + 1 g NaCl + 30 mL water	8.0 ± 0.16	1.253 ± 0.01	6.2 ± 0.11	3 h 30 min	8.0 ± 0.24	0.2987 ± 0.02	5.48 ± 0.39
D	Compressed fresh yeast	1 g yeast + 50 g flour + 1 g NaCl + 30 mL water	8.0 ± 0.30	1.253 ± 0.01	6.2 ± 0.11	3 h 30 min	8.0 ± 0.27	0.2948 ± 0.08	5.47 ± 0.37
F	Active dry yeast	3.5 g yeast + 50 g flour + 1 g NaCl + 30 mL water	7.3 ± 0.21	1.253 ± 0.01	6.2 ± 0.11	4 h 30 min	7.4 ± 0.28	0.3064 ± 0.07	5.62 ± 0.40
G	Active dry yeast	3 g yeast + 50 g flour + 1 g NaCl + 30 mL water	5.0 ± 0.21	1.253 ± 0.01	6.2 ± 0.11	7 h 30 min	5.0 ± 0.99	0.2078 ± 0.03	5.72 ± 0.16
H	Dried yeast	0.7 g yeast + 50 g flour + 1 g NaCl + 30 mL water	7.1 ± 0.35	1.253 ± 0.01	6.2 ± 0.11	7 h 30 min	7.3 ± 0.23	0.5431 ± 0.09	5.71 ± 0.08

**Table 3:** Microbiological and physicochemical characteristics of doughs at time zero (T0, after inoculation) and at the end of leavening using different starters. Doughs were analyzed for LAB and yeast counts (Log CFU/g), pH, total titratable acidity (TTA), and leavening time. The fructan content in the final model-doughs (g/100 g) was also measured.

	Time 0 (Inoculation)			End of Leavening					
Dough	Starter	LAB Log CFU/g	Yeasts Log CFU/g	LAB Log CFU/g	Yeasts Log CFU/g	pH	TTA	Leavening Time	Fructan Level g/100 g
A	Y	2.20 ± 0.25 ^a^	5.80 ± 0.28 ^a^	2.5 ± 0.06 ^a^	6.88 ± 0.00 ^a^	5.48 ± 0.02 ^d^	2.05 ± 0.07 ^bc^	4 h 30 min	0.2862 ± 0.0041 ^g^
B	Y	2.30 ± 0.14 ^a^	7.30 ± 0.15 ^b^	2.6 ± 0.07 ^a^	6.85 ± 0.01 ^a^	5.41 ± 0.04 ^d^	2.40 ± 0.14 ^ab^	3 h 40 min	0.3011 ± 0.0093 ^g^
C	Y	2.10 ± 0.13 ^a^	8.08 ± 0.18 ^cd^	2.8 ± 0.13 ^a^	7.85 ± 0.16 ^a^	5.38 ± 0.04 ^d^	2.80 ± 0.00 ^a^	3 h 40 min	0.2193 ± 0.0017 ^c^
D	LA1 + Y	9.30 ± 0.23 ^bc^	8.18 ± 0.11 ^cd^	8.68 ± 0.16 ^b^	7.85 ± 0.11 ^a^	4.68 ± 0.08 ^bc^	3.60 ± 0.14 ^de^	3 h 40 min	0.2267 ± 0.0020 ^d^
E	LA2 + Y	9.20 ± 0.11 ^bc^	8.02 ± 0.08 ^cd^	8.8 ± 0.14 ^bc^	7.81 ± 0.56 ^a^	4.51 ± 0.01 ^bc^	3.20 ± 0.00 ^cd^	3 h 40 min	0.2491 ± 0.0036 ^e^
F	LA1 + LA2 + Y	8.72 ± 0.05 ^b^	5.10 ± 0.14 ^a^	9.09 ± 0.37 ^bc^	7.65 ± 0.14 ^a^	4.32 ± 0.04 ^b^	4.00 ± 0.14 ^ef^	4 h 30 min	0.2602 ± 0.0011 ^f^
G	LA1 + LA2 + Y	8.60 ± 0.30 ^b^	7.46 ± 0.21 ^bc^	8.88 ± 0.32 ^bc^	6.90 ± 0.54 ^a^	4.68 ± 0.11 ^c^	4.80 ± 0.28 ^g^	3 h 40 min	0.2007 ± 0.0032 ^b^
H	LA1 + LA2 + Y	8.57 ± 0.32 ^b^	8.23 ± 0.25 ^d^	8.78 ± 0.28 ^bc^	7.85 ± 0.00 ^a^	4.77 ± 0.19 ^c^	4.00 ± 0.14 ^ef^	3 h 40 min	0.2676 ± 0.0062 ^f^
I	LA1 + LA2 + Y	9.66 ± 0.33 ^c^	8.28 ± 0.14 ^d^	9.6 ± 0.08 ^c^	7.60 ± 0.28 ^a^	3.84 ± 0.08 ^a^	5.60 ± 0.28 ^h^	3 h 40 min	0.1747 ± 0.0032 ^a^

Abbreviations: Y, commercial baker’s yeast (*Saccharomyces cerevisiae*); LA1 and LA2, lactic acid bacteria strains. All strains were selected as described in Section 3.3. Different letters in a column mean significant differences between samples (*p* < 0.05).

**Table 4:** Microbiological and physicochemical characteristics of doughs at time zero (T0, after inoculation) and at the end of leavening using different starters. Doughs were analyzed for LAB and yeast counts (Log CFU/g), pH, total titratable acidity (TTA), glucose, fructose, mannitol and fructan content.

Time 0 (Inoculation)				End of leavening							
Dough	Starter	LAB Log CFU/g	Yeasts Log CFU/g	LAB Log CFU/g	Yeasts Log CFU/g	Glucose (g/100 g)	Fructose (g/100 g)	Mannitol (g/100 g)	Fructan (g/100 g)	pH	TTA
C	Y	2.20 ± 0.00 ^a^	8.38 ± 0.20 ^a^	2.50 ± 0.07 ^a^	8.04 ± 0.15 ^a^	0.021 ± 0.004 ^a^	0.065 ± 0.009 ^b^	0.005 ± 0.002 ^b^	0.3098 ± 0.006 ^b^	5.11 ± 0.15 ^b^	2.4 ± 0.0 ^a^
H	LA1 + LA2 + Y	8.38 ± 0.28 ^b^	8.36 ± 0.35 ^a^	8.25 ± 0.00 ^b^	8.08 ± 0.53 ^a^	0.015 ± 0.003 ^a^	0.060 ± 0.006 ^b^	0.000 ± 0.000 ^a^	0.3250 ± 0.006 ^c^	4.84 ± 0.21 ^b^	2.8 ± 0.2 ^a^
I	LA1 + LA2 + Y	9.10 ± 0.11 ^c^	8.40 ± 0.12 ^a^	9.23 ± 0.00 ^c^	8.30 ± 0.42 ^a^	0.013 ± 0.006 ^a^	0.020 ± 0.003 ^a^	0.000 ± 0.000 ^a^	0.2323 ± 0.015 ^a^	4.29 ± 0.13 ^a^	4.4 ± 0.4 ^b^

Abbreviations: Y, commercial baker’s yeast (*Saccharomyces cerevisiae*); LA1 and LA2, lactic acid bacteria strains. Different letters in a column mean significant differences between samples (*p* < 0.05).

## Data Availability

The original contributions presented in this study are included in the article/Supplementary Materials. Further inquiries can be directed to the corresponding authors.

## Supplementary Materials

The following supporting information can be downloaded at: https://www.mdpi.com/article/10.3390/foods15162792/s1, Table S1: Lactic acid bacteria strains used in the study.

## Abbreviations

The following abbreviations are used in this manuscript:

CFU	Colony Forming Unit
FODMAPs	Fermentable Oligosaccharides, Disaccharides, Monosaccharides, And Polyols
FOS	Fructo-oligosaccharides
GLU/FRU	Glucose/Fructose
GOS	Galacto-oligosaccharides
IBS	Irritable Bowel Syndrome
LAB	Lactic Acid Bacteria
TTA	Total Titratable Acidity
W	Dough Strength
