# Supplementary material for: Integrated Strategy for Low-FODMAP Bread Formulation: Selection of Wheat Flours and Microbial Starter
Source: Foods. 2026 Aug 9;15(16):2792. doi: 10.3390/foods15162792 (PMC13512153; doi:10.3390/foods15162792)
Supplement: Supplementary file 1 [file foods-15-02792-s001.zip › foods-4454606-supplementary.pdf]

Tab. S1 Lactic acid bacteria strains used in the study

N.	Strain	Species	Source
**1**	ISACC 1001	*Companilactobacillus paralimentarius*	Sourdough
**2**	ISACC 1003	*Lactiplantibacillus pentosus-plantarum*	Sourdough
**3**	ISACC 1004	*Lactiplantibacillus plantarum*	Sourdough
**4**	ISACC 1005	*Lactiplantibacillus plantarum - pentosus*	Sourdough
**5**	ISACC 1007	*Lactiplantibacillus plantarum*	Sourdough
**6**	ISACC 1008	*Furfurilactobacillus rossiae*	Sourdough
**7**	ISACC 1011	*Companilactobacillus paralimentarius*	Sourdough
**8**	ISACC 1012	*Levilactobacillus brevis*	Sourdough
**9**	ISACC 1013	*Pediococcus pentosaceus*	Sourdough
**10**	ISACC 1014	*Lactiplantibacillus plantarum*	Sourdough
**11**	ISACC 1015	*Levilactobacillus brevis*	Sourdough
**12**	ISACC 1016	*Lactiplantibacillus plantarum*	Sourdough
**13**	ISACC 1018	*Lactiplantibacillus plantarum*	Sourdough
**14**	ISACC 1019	*Pediococcus acidilactici*	Sourdough
**15**	ISACC 1020	*Levilactobacillus brevis*	Sourdough
**16**	ISACC 1021	*Latilactobacillus sakei*	Sourdough
**17**	ISACC 1026	*Levilactobacillus brevis*	Sourdough
**18**	ISACC 1028	*Lactiplantibacillus plantarum*	Sourdough
**19**	ISACC 1030	*Latilacrobacillus curvatus*	Sourdough
**20**	ISACC 1031	*Weissella cibaria*	Sourdough
**21**	ISACC 1032	*Levilactobacillus brevis*	Sourdough
**22**	ISACC 1040	*Leuconostoc mesenteroides-pseudomesenteroides*	Sourdough
**23**	ISACC 1041	*Lactiplantibacillus plantarum*	Sourdough
**24**	ISACC 1045	*Leuconostoc mesenteroides-pseudomesenteroides*	Sourdough
**25**	ISACC 1046	*Weissella cibaria*	Sourdough
**26**	ISACC 1047	*Furfurilactobacillus rossiae*	Sourdough
**27**	ISACC 1051	*Lactiplantibacillus plantarum*	Sourdough
**28**	ISACC 1052	*Furfurilactobacillus rossiae*	Sourdough
**29**	ISACC 1053	*Pediococcus petosaceus*	Sourdough
**30**	ISACC 1055	*Furfurilactobacillus rossiae*	Sourdough
**31**	ISACC 1056	*Lactiplantibacillus plantarum*	Sourdough
**32**	ISACC 1057	*Furfurilactobacillus rossiae*	Sourdough
**33**	ISACC 1060	*Furfurilactobacillus rossiae*	Sourdough
**34**	ISACC 1061	*Furfurilactobacillus rossiae*	Sourdough
**35**	ISACC 1064	*Companilactobacillus paralimentarius*	Sourdough
**36**	ISACC 1065	*Lactiplantibacillus plantarum*	Sourdough
**37**	ISACC 1067	*Furfurilactobacillus rossiae*	Sourdough
**38**	ISACC 1068	*Weissella cibaria*	Sourdough
**39**	ISACC 1070	*Lacticaseibacillus paracasei-casei*	Sourdough
**40**	ISACC 1071	*Lacticaseibacillus paracasei-casei*	Sourdough
**41**	ISACC 1074	*Pediococcus pentosaceus*	Sourdough
**42**	ISACC 1075	*Pediococcus pentosaceus*	Sourdough
**43**	ISACC 1076	*Pediococcus pentosaceus*	Sourdough
**44**	ISACC 1077	*Pediococcus pentosaceus*	Sourdough
**45**	ISACC 1078	*Leuconostoc mesenteroides*	Sourdough
**46**	ISACC 1079	*Lactiplantibacillus plantarum*	Sourdough
**47**	ISACC 1080	*Pediococcus pentosaceus*	Sourdough
**48**	ISACC 1081	*Levilactobacillus brevis*	Sourdough
**49**	ISACC 1082	*Pediococcus pentosaceus*	Sourdough
**50**	ISACC 1083	*Lactiplantibacillus plantarum*	Sourdough
**51**	ISACC 1084	*Levilactobacillus brevis*	Sourdough
**52**	ISACC 1085	*Lactiplantibacillus plantarum*	Sourdough
**53**	ISACC 1087	*Pediococcus pentosaceus*	Sourdough
**54**	ISACC 1088	*Lactiplantibacillus plantarum - pentosus*	Sourdough
**55**	ISACC 1089	*Furfurilactobacillus rossiae*	Sourdough
**56**	ISACC 1090	*Lactiplantibacillus plantarum*	Sourdough
**57**	ISACC 1091	*Latilacrobacillus curvatus*	Sourdough
**58**	ISACC 1092	*Levilactobacillus brevis*	Sourdough
**59**	ISACC 1093	*Latilactobacillus curvatus*	Sourdough
**60**	ISACC 1094	*Latilactobacillus curvatus*	Sourdough
**61**	ISACC 1095	*Lactiplantibacillus plantarum*	Sourdough
**62**	ISACC 1096	*Lactiplantibacillus plantarum*	Sourdough
**63**	ISACC 1097	*Lactiplantibacillus plantarum*	Sourdough
**64**	ISACC 1098	*Lactiplantibacillus plantarum-argentoratensis-pentosus*	Sourdough
**65**	ISACC 1099	*Pediococcus pentosaceus*	Sourdough
**66**	ISACC 1100	*Leuconostoc lactis*	Sourdough
**67**	ISACC 1101	*Pediococcus pentosaceus*	Sourdough
**68**	ISACC 1102	*Leuconostoc lactis*	Sourdough
**69**	ISACC 1103	*Leuconostoc lactis*	Sourdough
**70**	ISACC 1104	*Lactiplantibacillus plantarum*	Sourdough
**71**	ISACC 1106	*Lactiplantibacillus plantarum*	Sourdough
**72**	ISACC 1107	*Pediococcus pentosaceus*	Sourdough
**73**	ISACC 1108	*Pediococcus pentosaceus*	Sourdough
**74**	ISACC 1110	*Pediococcus pentosaceus*	Sourdough
**75**	ISACC 1111	*Weissella cibaria*	Sourdough
**76**	ISACC 1112	*Lacticaseibacillus paracasei-casei*	Sourdough
**77**	ISACC 1113	*Lactiplantibacillus plantarum-argentoratensis-pentosus*	Sourdough
**78**	ISACC 1114	*Pediococcus pentosaceus*	Sourdough
**79**	ISACC 1115	*Levilactobacillus brevis*	Sourdough
**80**	ISACC 1117	*Lactiplantibacillus plantarum*	Sourdough
**81**	ISACC 1118	*Levilactobacillus brevis*	Sourdough
**82**	ISACC 1119	*Levilactobacillus brevis*	Sourdough
**83**	ISACC 1121	*Companilactobacillus paralimentarius*	Sourdough
**84**	ISACC 1122	*Companilactobacillus paralimentarius*	Sourdough
**85**	ISACC 1124	*Companilactobacillus paralimentarius*	Sourdough
**86**	ISACC 1125	*Pediococcus pentosaceus*	Sourdough

**87**	ISACC 1127	*Companilactobacillus paralimentarius*	Sourdough
**88**	ISACC 1128	*Lactiplantibacillus plantarum*	Sourdough
**89**	ISACC 1129	*Lactiplantibacillus plantarum*	Sourdough
**90**	ISACC 1130	*Levilactobacillus brevis*	Sourdough
**91**	ISACC 1131	*Lactiplantibacillus plantarum*	Sourdough
**92**	ISACC 1132	*Leuconostoc mesenteroides-pseudomesenteroides*	Sourdough
**93**	ISACC 1134	*Lactiplantibacillus plantarum*	Sourdough
**94**	ISACC 1135	*Companilactobacillus paralimentarius-kimchi*	Sourdough
**95**	ISACC 1140	*Lactiplantibacillus paraplantarum-plantarum*	Sourdough
**96**	ISACC 1142	*Lactiplantibacillus pentosus-L. plantarum-argentoratensis*	Sourdough
**97**	ISACC 1144	*Levilactobacillus brevis*	Sourdough
**98**	ISACC 1145	*Levilactobacillus brevis*	Sourdough
**99**	ISACC 1146	*Lactiplantibacillus plantarum*	Sourdough
**101**	ISACC 1148	*Lactiplantibacillus plantarum-argentoratensis*	Sourdough
**102**	ISACC 1150	*Lactiplantibacillus plantarum*	Sourdough
**103**	ISACC 1152	*Lactiplantibacillus plantarum-argentoratensis-pentosus*	Sourdough
**104**	ISACC 1155	*Lactiplantibacillus plantarum*	Sourdough
**105**	ISACC 1156	*Furfurilactobacillus rossiae*	Sourdough
**106**	ISACC 1158	*Furfurilactobacillus rossiae*	Sourdough
**107**	ISACC 1159	*Levilactobacillus brevis*	Sourdough
**108**	ISACC 1164	*Furfurilactobacillus rossiae*	Sourdough
**109**	ISACC 1168	*Levilactobacillus brevis*	Sourdough
**110**	ISACC 1171	*Levilactobacillus brevis*	Sourdough
**111**	ISACC 1178	*Pediococcus pentosaceus*	Sourdough
**112**	ISACC 1179	*Furfurilactobacillus rossiae*	Sourdough
**113**	ISACC 1180	*Levilactobacillus brevis*	Sourdough
**114**	ISACC 1181	*Furfurilactobacillus rossiae*	Sourdough
**115**	ISACC 1182	*Furfurilactobacillus rossiae*	Sourdough
**116**	ISACC 1188	*Furfurilactobacillus rossiae*	Sourdough
**117**	ISACC 1191	*Levilactobacillus brevis*	Sourdough
**118**	ISACC 1229	*Pediococcus acidilactici*	Sourdough
**119**	ISACC 1234	*Furfurilactobacillus rossiae*	Sourdough
**120**	ISACC 1236	*Furfurilactobacillus rossiae*	Sourdough
**121**	ISACC 1237	*Furfurilactobacillus rossiae*	Sourdough
**122**	ISACC 1238	*Furfurilactobacillus rossiae*	Sourdough
**123**	ISACC 1240	*Lactiplantibacillus plantarum*	Sourdough
**124**	ISACC 1255	*Leuconostoc lactis*	Sourdough
**125**	ISACC 1256	*Leuconostoc lactis*	Sourdough
**126**	ISACC 1257	*Leuconostoc lactis*	Sourdough
**127**	ISACC 1258	*Leuconostoc lactis*	Sourdough
**128**	ISACC 1259	*Leuconostoc lactis*	Sourdough
**129**	ISACC 1260	*Lacticaseibacillus rhamnosus*	Sourdough
**130**	ISACC 1261	*Lactiplantibacillus plantarum*	Sourdough
**131**	ISACC 1262	*Pediococcus pentosaceus*	Sourdough
**132**	ISACC 1264	*Leuconostoc lactis*	Sourdough
**133**	ISACC 1265	*Leuconostoc lactis*	Sourdough
**134**	ISACC 1266	*Leuconostoc lactis*	Sourdough
**135**	ISACC 1267	*Leuconostoc lactis*	Sourdough
**136**	ISACC 1268	*Leuconostoc lactis*	Sourdough
**137**	ISACC 1269	*Pediococcus pentosaceus*	Sourdough
**138**	ISACC 1270	*Lactiplantibacillus plantarum*	Sourdough
**139**	ISACC 1276	*Leuconostoc lactis*	Sourdough
**140**	ISACC 1277	*Leuconostoc lactis*	Sourdough
**141**	ISACC 1278	*Leuconostoc lactis*	Sourdough
**142**	ISACC 1280	*Lactiplantibacillus plantarum*	Sourdough
**143**	ISACC 1281	*Lactiplantibacillus plantarum*	Sourdough
**144**	ISACC 1283	*Lactiplantibacillus plantarum*	Sourdough
**145**	ISACC 1284	*Leuconostoc lactis*	Sourdough
**146**	ISACC 1286	*Leuconostoc lactis*	Sourdough
**147**	ISACC 1287	*Lactococcus lactis*	Sourdough
**148**	ISACC 1289	*Lactococcus lactis*	Sourdough
**149**	ISACC 1292	*Lactiplantibacillus plantarum*	Sourdough
**150**	ISACC 1293	*Lactiplantibacillus plantarum*	Sourdough
